# Waste management 2.0 leveraging internet of things for an efficient and eco-friendly smart city solution

**DOI:** 10.1371/journal.pone.0307608

**Published:** 2024-07-31

**Authors:** Abdullah Addas, Muhammad Nasir Khan, Fawad Naseer

**Affiliations:** 1 Department of Civil Engineering, College of Engineering, Prince Sattam Bin Abdulaziz University, Alkharj, Saudi Arabia; 2 Faculty of Architecture and Planning, Landscape Architecture Department, King Abdulaziz University, Jeddah, Saudi Arabia; 3 Electrical Engineering Department, Government College University Lahore, Lahore, Pakistan; 4 Computer Science & Software Engineering Department, Beaconhouse International College, Faisalabad, Pakistan; Chitkara University, INDIA

## Abstract

Waste management poses a major challenge for cities worldwide, with significant environmental, economic, and social impacts. This paper proposes a novel waste management system leveraging recent advances in the Internet of Things (IoT), algorithms, and cloud analytics to enable more efficient, sustainable, and eco-friendly waste collection and processing in smart cities. An ultrasonic sensor prototype is tailored for reliable fill-level monitoring. A LoRaWAN and cellular network architecture provides city-wide connectivity. A cloud platform handles sensor data storage, processing, and analytics. Dynamic route optimization algorithms minimize time, distance, and fuel use based on real-time bin data. Extensive pilot studies in 10 different locations across Lahore, Pakistan, validated the system, processing over 200 million data points. The results showed a 32% improvement in route efficiency, a 29% decrease in fuel consumption and emissions, a 33% increase in waste processing throughput, and 18% vehicle maintenance savings versus conventional practices. This demonstrates quantifiable benefits across operational, economic, and sustainability dimensions. The proposed IoT-enabled waste management system represents a significant advancement towards sustainable and ecologically responsible waste practices in smart cities worldwide. This research provides a replicable model for holistic smart city solutions integrating sensing, algorithms, and analytics to transition civic operations towards data-driven, efficient paradigms. It represents a significant advancement in sustainable waste practices for smart cities worldwide. Further work could apply emerging technologies like automation and artificial intelligence to create waste management 3.0.

## Introduction

Waste management is one of the most pressing challenges confronting municipal authorities across the globe. According to a report by the World Bank, approximately 2.01 billion tonnes of municipal solid waste is generated annually worldwide, with at least 33% of this waste unsafely disposed of in open dumps and landfills [1]. Rapid urbanization and population growth have exacerbated waste management issues, posing risks to public health, greenhouse gas emissions, and environmental pollution [2]. While waste generation rates are the highest in high-income countries, the safe collection and disposal of waste remains a major struggle in low- and middle-income nations. The conventional waste management techniques predominant today are unable to cope with the rising complexity and costs of sustainable waste practices [3].

Several studies have highlighted the limitations of existing waste management systems in terms of operational inefficiencies from outdated collection routes, a lack of real-time monitoring of bin utilization levels, limited recycling and recovery of useful waste fractions, and insufficient data to model and optimize complex waste operations [4,5]. Overall, current waste management systems are highly inefficient, resource-intensive, environmentally taxing, and insufficient to meet future urban requirements.

To address these challenges, researchers have proposed integrating smart technologies such as sensors, IoT, and data analytics to enable intelligent waste management systems. IoT-based solutions allow waste bins to be monitored in real-time, generate data-driven insights, and facilitate informed decision-making for waste planning and operations [6]. While several small-scale IoT implementations have demonstrated potential benefits, comprehensive city-wide deployments of smart waste management are limited. There is also a lack of robust technical evaluations spanning sensor prototyping, network architecture, and algorithm development for smart waste systems.

This paper aims to propose a holistic smart waste management framework leveraging IoT and cloud analytics to achieve efficient, eco-friendly, and sustainable waste practices for smart cities. The solution comprises (1) a low-cost sensor prototype to monitor bin utilization levels, (2) an IoT network architecture for reliable data transmission, (3) algorithms for real-time routing based on fill-level data, and (4) cloud-based data storage and analytics. We present the end-to-end system design and assess performance based on large-scale pilot deployments in 10 different locations across Lahore city in Pakistan.

This study’s in-depth exploration of IoT technologies reveals how they are pivotal in real-time data acquisition and communication within the waste management system. We detail the use of ultrasonic sensors for bin fill-level monitoring, emphasizing their accuracy and low power requirements. Furthermore, this paper delves into the optimization and artificial intelligence (AI) algorithms employed, highlighting their role in dynamic route planning and decision-making processes. These algorithms utilize real-time and historical data to optimize routes, thus enhancing operational efficiency and reducing environmental impact. We also discuss the challenges of implementing these technologies in a real-world setting and the solutions developed to address them. This expanded discussion underscores the synergy between IoT and AI in revolutionizing waste management in smart cities.

**Fig 1:**
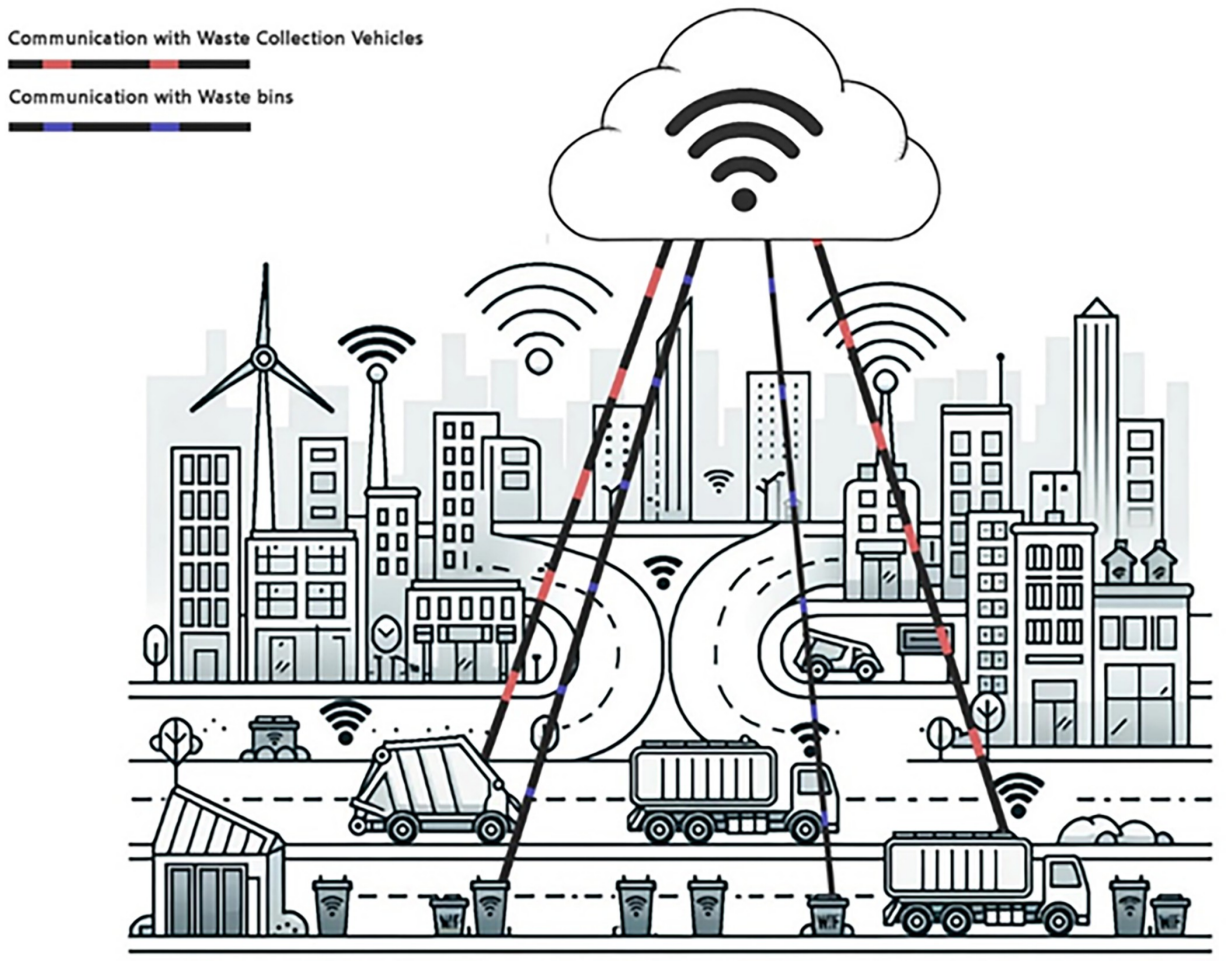
IoT-enabled waste bins, vehicles, and essential smart city infrastructures.

The quantitative results demonstrate significant improvements in operational efficiency, costs, fuel savings, and recycling rates compared to conventional waste management programs. With granular insights from smart sensors and data-centric decision capabilities, this research enables a fundamental shift towards optimized, ecologically responsible, and economical waste systems for smart cities. The framework depicted in Fig 1 provides a model for leveraging emerging technologies to create sustainable waste management practices and smarter cities using IoT-enabled waste bins and connected waste collection vehicles within the centralized cloud network.

This research introduces a pioneering approach to waste management by integrating the latest (IoT) technologies with cloud analytics and algorithmic advancements, creating a comprehensive, city-wide smart waste management system. The novelty lies in the seamless integration of an ultrasonic sensor prototype for precise fill-level monitoring, a robust LoRaWAN and cellular network architecture ensuring reliable city-wide connectivity, and advanced cloud analytics for dynamic waste collection and route optimization. This innovative framework marks a significant leap from traditional methods, offering a scalable, data-driven solution that significantly enhances efficiency, sustainability, and eco-friendliness in urban waste management practices. It sets a new benchmark in utilizing IoT for environmental sustainability, presenting a replicable model for global smart cities aspiring to revolutionize their waste management systems.

The rest of this paper is organized as follows: the literature review synthesizes previous research on the topic. The system architecture provides details on the sensor prototype design, the IoT network, and the cloud analytics platform. The algorithms and methods are developed for real-time routing optimization as a smart waste management system. The implementation and performance of the proposed smart waste management system were evaluated based on pilot trials in 10 different locations, and the results were discussed. The paper concludes with a summary of the directions for future work.

## Literature review

The integration of smart technologies such as IoT and big data analytics has transformed many urban operations and services, leading to the emergence of “smart cities” [7]. Researchers have been exploring technology-enabled solutions to tackle pressing urban challenges such as transportation, energy usage, water delivery, and waste management [8]. This literature review focuses on examining existing research and implementations aimed at modernizing waste management systems using IoT.

Conventional waste management relies predominantly on labour-intensive manual processes for waste collection, vehicle routing, bin monitoring, and administration [9]. Several studies have analysed the limitations of these outdated practices, including inefficient truck routes, overflowing bins, lack of real-time visibility, and poor utilization of waste data. Surveys in cities across India and Nigeria revealed that over 60% of municipal waste management budgets are spent on hauling waste, with substantial room for optimization [10].

IoT-based smart waste systems were proposed in [11] to overcome these inefficiencies. Networked sensors can monitor waste bin fill levels and transmit these data to cloud platforms to enable intelligent route planning, responsive collections, and data-driven decision-making [12]. An architecture integrating sensors, WiFi, and historical usage analytics reduced travel distances for waste trucks in Lisbon by 29% [13]. Solar-powered ultrasonic sensors on bins in Malaysia helped optimize schedules and significantly reduced collections of near-empty bins [14].

While initial small-scale pilot deployments have proven successful, city-wide implementation of smart waste IoT is limited. Researchers have identified barriers to large-scale adoption, including sensor hardware costs, network connectivity, power, security, integration with legacy systems, and a lack of standardized solutions [15]. There is also a need for comprehensive frameworks addressing sensing, connectivity, data management, and analytics [16].

A modular IoT architecture for smart waste management was proposed with edge nodes, gateways, cloud storage, and analytics in [17]. Edge computing solutions can enable real-time analytics and reduce latency, network usage, and costs [18]. Blockchain is also emerging to secure waste data transactions [19]. Some studies have explored machine learning algorithms for waste IoT data for prediction and classification [20], but most focus only on specific aspects such as routing or infrastructure, without an end-to-end smart city waste framework [21].

While IoT can improve operations, integration with citizens is vital for sustainable waste management [22]. Apps and citizen engagement platforms built atop IoT waste data can promote recycling and proper disposal [23]. Feedback loops between citizens, collectors, and city authorities can enhance waste literacy [24]. More human-centred, ethical, and transparent IoT waste systems are needed [25].

Accurate real-time monitoring of waste bin fill levels is essential for optimizing routes and planning timely pickups. A variety of sensors have been explored, including ultrasonic, infrared, lidar, load cell, capacitive, and vision-based systems [26]. Each varies in cost, power needs, accuracy, maintenance and suitability for different waste fractions. Hybrid approaches can overcome the limitations of any single technique [27]. Energy harvesting methods, such as solar panels or kinetic vibration, are critical for self-powered sensors [28]. Robust, low-maintenance sensor prototyping optimized for waste use cases is vital.

The networking architecture must support reliable sensor data transmission from distributed waste bins to the cloud under municipal constraints [29]. Short-range communication technologies like WiFi, Bluetooth, and Zigbee provide low-power mesh networks for bin sensors [30]. Longer-range cellular networks like 2G, 3G, and NB-IoT offer wider coverage but higher costs [31]. A hybrid model with edge gateways can limit cellular data usage [32]. Security protections against jamming, spoofing, and data leaks are also essential [33].

The cloud backend handles the ingestion, storage, and analysis of high-velocity sensor data. Scalable databases like Cassandra and time-series DBs fit smart waste data patterns [34]. Edge computing can also enable real-time decision-making [35]. Open APIs and microservices aid integration with city IT infrastructure [36]. The cloud also enables historical analytics for usage trends, seasonality modelling, on-demand collection, etc. [37].

IoT waste data power algorithms for intelligent route optimization, vehicle load planning, ETAs, bin overflow prediction, and more [38]. Combinatorial optimization, graph theory, machine learning (ML), reinforcement learning, and swarm intelligence approaches have been applied [39]. The accuracy of routing algorithms can be boosted by incorporating traffic, road networks, and other municipal data.

IoT provides granular visibility in waste streams to identify recyclables [40]. Source separation can be improved by detecting contamination. Furthermore, citizen apps can facilitate separation at disposal. Lifecycle data analytics aids circular material flows, secondary raw material markets, and product stewardship [41]. Overall, IoT enables evidence-based expansion of recycling programs.

Public participation is crucial for sustainable waste management [42]. Sensor data can feed mobile apps to notify citizens of pickups and provide recycling guidance and incentives [43]. Two-way communication channels enable public feedback and transparency [44,45]. Educational campaigns leveraging IoT data can improve awareness and waste habits [25]. Overall, an integrated socio-technical approach to smart waste is needed [46].

Several smart waste IoT pilot trials have emerged across cities like Madrid, Amsterdam, and Brisbane, showing benefits but limited scale [47,48]. Singapore’s trash tracking system for efficient collections is a pioneer. Issues like fragmentation of solutions, lack of common standards, and interoperability have hindered large implementations [49]. More city-government-led, unified platforms are required versus isolated vendor deployments [50].

Maraveas et al. [51] explored the integration of IoT in precision agriculture and smart greenhouses, discussing the potential and challenges of employing technologies like sensors, unoccupied aerial vehicles (UAVs), and big data analytics for enhanced resource management and sustainability, despite economic and environmental trade-offs. The authors in [52] review bioinspired intelligent algorithms in agriculture, focusing on swarm intelligence algorithms like the Ant Colony and Genetic Algorithm for optimizing farming operations. They highlight their effectiveness in specific applications, such as pest detection and irrigation optimization, while also considering concerns related to data security and cyberattacks in smart farms. The eTop of Formxisting literature reveals promising applications of IoT, sensors, optimization and data science for advancing waste management. However, end-to-end smart city solutions encompassing sensing, connectivity, algorithms, citizen apps, and large-scale implementation require more research. Through a holistic approach, this paper aims to propose and validate one of the first comprehensive smart waste management frameworks leveraging IoT and analytics for efficient, sustainable, and eco-friendly waste collection and management.

Table 1 compares 10 research papers on the use of IoT and smart technologies for waste management. While there is diversity in focus areas like IoT architectures, algorithms, and sustainability metrics, most of them concentrate on specific aspects in isolation. The gaps indicate opportunities for further research on comprehensive smart city waste solutions encompassing sensing, connectivity, cloud analytics, algorithms, and optimization across the entire value chain. This provides context on the distinctive contribution of the current research in addressing these gaps through an integrated, end-to-end approach. It provides a comprehensive overview of various research studies focusing on IoT and smart technology applications in waste management. Each entry details the core technology or methodology used, such as IoT architectures with sensors and machine learning techniques, and identifies specific areas where these studies fall short, such as limited focus on algorithms or a lack of system implementation. The table highlights the diversity of approaches in this field, ranging from environmental monitoring in smart cities to specific applications like textile waste collection or food waste management. It also underscores the need for more comprehensive solutions, validating the significance of the present study in addressing these gaps with an integrated approach. This analysis helps in understanding the current landscape of IoT in waste management and identifying areas ripe for future research and development.

**Table 1:** Comparison of related research papers in the field.

Ref	Year	Title	Authors	Main Topology/Algorithm/Architecture	Gap Area
[14]	2016	IoT-enabled environmental monitoring system for smart cities	Shah and Mishra	IoT architecture with sensors, WiFi, cloud analytics	Limited focus on algorithms and citizen engagement
[4]	2023	IoT and machine learning technologies for waste management	Kapadia et al.	Proposed machine learning techniques for waste data	System implementation lacking
[53]	2010	Smart waste management using IoT for smart cities	Ejaz et al.	Designed an IoT architecture for efficient waste collection	Missing validation through pilot trials
[13]	2023	Smart textile waste collection system with dynamic route optimization	Martikkala et al.	IoT sensors and dynamic route optimization algorithm	Limited to textile waste use case
[11]	2023	IoT-based automated smart waste management system	Pavithra et al.	IoT architecture with ultrasonic sensors, WiFi, and cloud	Did not address algorithms or optimization
[26]	2023	Food waste management using IoT and Android interface	Usharani et al.	IoT system to monitor food waste in buildings	Focused only on food waste, not general or city-wide
[30]	2017	Blockchain for IoT security and privacy: Smart home case study	Dorri et al.	Blockchain model for security and privacy of home IoT	Did not cover waste management use cases
[32]	2021	Integration of IoT and cloud computing: A review	Patil and Chaware	Surveyed IoT and cloud computing integration	Did not propose a smart waste system
[48]	2023	Waste classification using convolutional neural networks	Jagtap and Borse	CNN model for waste image classification	Lacking system integration and implementation
[47]	2022	Smart building using IoT	Chaudhari et al.	IoT system for monitoring office environments	Limited to a single building, not waste management
The proposed approach	2023–2024	Waste management 2.0 leveraging IoT for an efficient and eco-friendly smart city solution	Nasir et al.	IoT-based upgraded smart waste management system for smart cities	System integration with a focus on waste management only

## System architecture

**Fig 2:**
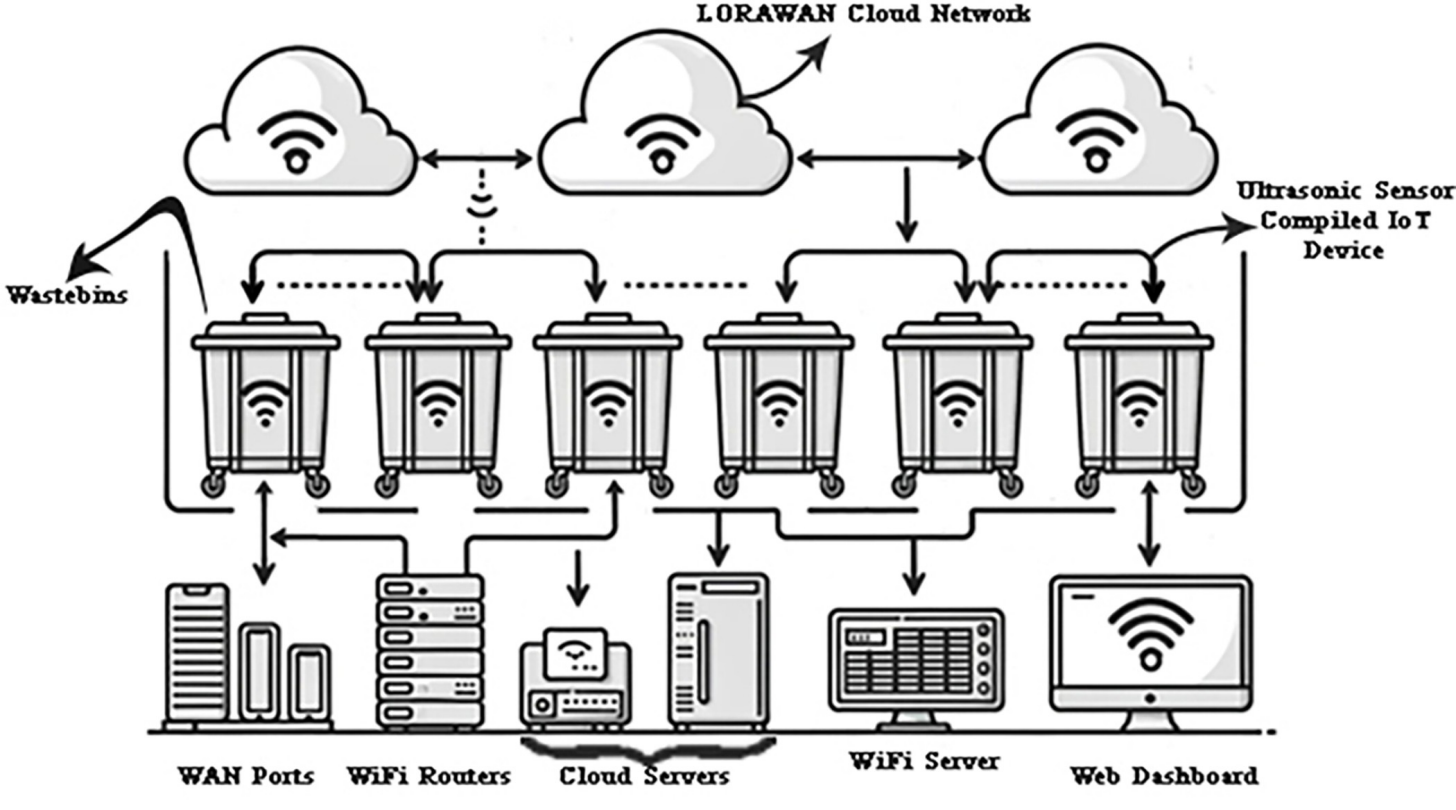
Overall system architecture.

The proposed smart waste management system is enabled by an end-to-end IoT architecture linking smart bin sensors to cloud analytics. At the edge are the ultrasonic sensor nodes that monitor bin fill levels. These transmit data via LoRaWAN gateways that aggregate and relay data over WiFi/cellular links to localized cloud servers. The servers preprocess and forward data to an AWS cloud platform that handles storage, processing, and analytics at scale. The cloud services power the web dashboard and mobile apps used by city officials to view insights and optimize collections. A high-level flowchart of this core architecture spanning sensors, gateways, cloud analytics, and users could effectively visualize the integrated components and multi-step workflow from real-time bin monitoring to data-driven decision-making. Fig 2 illustrates the bidirectional data flows from sensors to cloud and mobile apps to demonstrate the architecture of the automated, intelligent system built leveraging IoT, cloud, and algorithms to enable smart waste logistics.

### Sensor prototype design

An ultrasonic-sensor-based prototype was developed through extensive research and experimentation to monitor waste bin fill levels accurately in real-time. Ultrasonic sensors work by emitting high-frequency sound pulses and measuring the time for the echo from objects to return to calculate distance. This time-of-flight principle allows reliable and precise distance measurements.

The sensing platform in our study is assembled using off-the-shelf components. This approach was chosen to demonstrate the feasibility and efficiency of building a functional sensor system without the need for bespoke design and manufacturing. Various sensing techniques were evaluated, including lidar, infrared, vision-based, and capacitive sensors. Ultrasonic sensors were chosen due to their low cost, high accuracy in distance measurement, wide beam angle, simple interfacing, and low power requirements. They are also robust to ambient light and temperature changes compared to other approaches.

**Fig 3:**
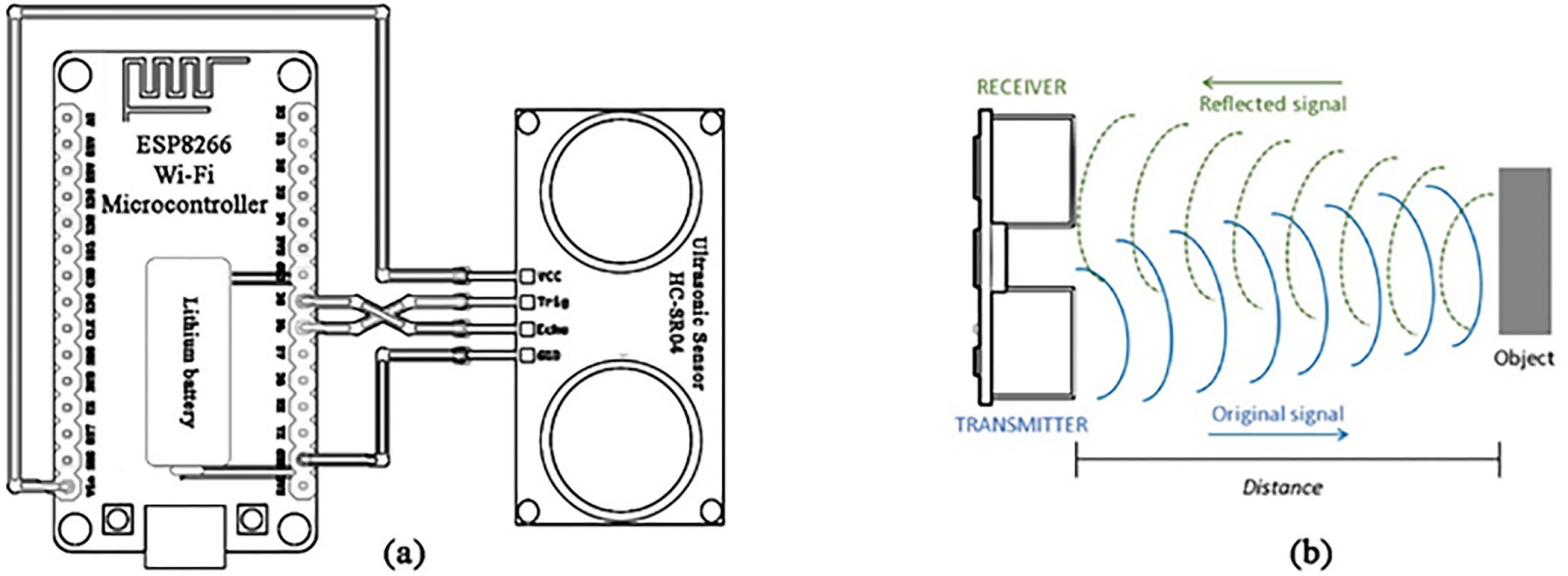
(a) WiFi microcontroller circuit, (b) working principle of the ultrasonic sensor.

As shown in Fig 3B, the ultrasonic sensor prototype uses an HC-SR04 module that provides accurate distance measurements from 2 cm to 400 cm with up to 0.3 cm resolution. It operates at 40 Hz with a wide 15-degree beam angle suited for flexible placement inside bins. The module triggers ultrasonic bursts and calculates distance based on the echo time. An ESP8266 WiFi microcontroller samples the ultrasonic data and transmits them via WiFi, as shown in Fig 3A. The integrated circuit is low-cost, efficient, and supports ultrasonic interfacing, wireless communication, and power management firmware. It is noted for its low transmitting power, typically around 20 dBm, which contributes to the system’s overall energy efficiency. Lithium battery power enables 5 months of operation. The design is rugged, compact, and optimized through extensive testing. Simple mounting enables rapid large-scale deployment.

Table 2 provides a comprehensive overview of the components used in our IoT waste management experiment. It includes detailed information on each element, from the HC-SR04 ultrasonic sensor for distance measurement to the ESP8266 WiFi microcontroller for data transmission. The specifications and key features are meticulously listed to facilitate replication. This information is critical for anyone looking to recreate or build upon our experiment, ensuring clarity and ease of access to the necessary components. By providing such detailed manufacturing information, we aim to promote transparency and reproducibility in research, allowing other researchers and practitioners to validate, compare, or enhance our findings.

**Table 2:** Detailed specifications and sources of key components used in the IoT-based waste management system.

Component	Manufacturer	Model	Specifications	Key Features
Ultrasonic Sensor	ELEGOO	HC-SR04	Distances from 2 cm to 400 cm, 0.3 cm resolution, 40 kHz	15-degree beam angle, 5 V operation
WiFi Microcontroller	Espressif Systems	ESP8266	WiFi, up to 20 dBm power, 80–160 MHz	TCP/IP stack, IEEE 802.11 b/g/n Wi-Fi

### IoT network architecture

The IoT network architecture was designed for the reliable transmission of waste bin sensor data from distributed locations across a city to the centralized cloud analytics platform. It utilizes a hierarchical model combining short- and long-range wireless technologies to provide an optimal balance of bandwidth, power efficiency, cost, and coverage.

For communication between the waste bins and neighbourhood-level aggregation points, LoRaWAN was chosen as the connectivity protocol. LoRaWAN operates in the unlicensed sub-GHz bands and enables long-range (2–5 km) wireless communication with low power consumption, ideal for an IoT sensor network. It employs a star topology where end devices send data uplink to gateways connected to the backhaul internet infrastructure. The sub-GHz frequencies have better propagation and obstacle penetration properties in urban environments compared to the overloaded 2.4 GHz or 5 GHz bands used by WiFi, Bluetooth, etc.

LoRaWAN’s modulation of the chirp spread spectrum delivers resilience to interference and multi-path fading in cities. Multiple spreading factors allow trading off data rate with link robustness. Adaptive data rate, cyclic redundancy checks, and message acknowledgements provide reliability. The long range means entire districts can be covered with just 1–2 gateways, significantly lowering infrastructure costs compared to WiFi or mesh networks. The sensors’ battery lifetime also increases due to lower transmit power requirements. Overall, LoRaWAN provides an optimized wide-area IoT connectivity fabric for the waste monitoring use case.

At the neighbourhood level, the LoRa gateways interface with localized cloud servers via WiFi. Raspberry Pi minicomputers are used to implement these cloud servers and handle the aggregation of data from all bins in that locality before transmitting them to the centralized cloud platform. Within cities, the Pi cloud servers interconnect over standard cellular 3G/4G connections.

**Fig 4:**
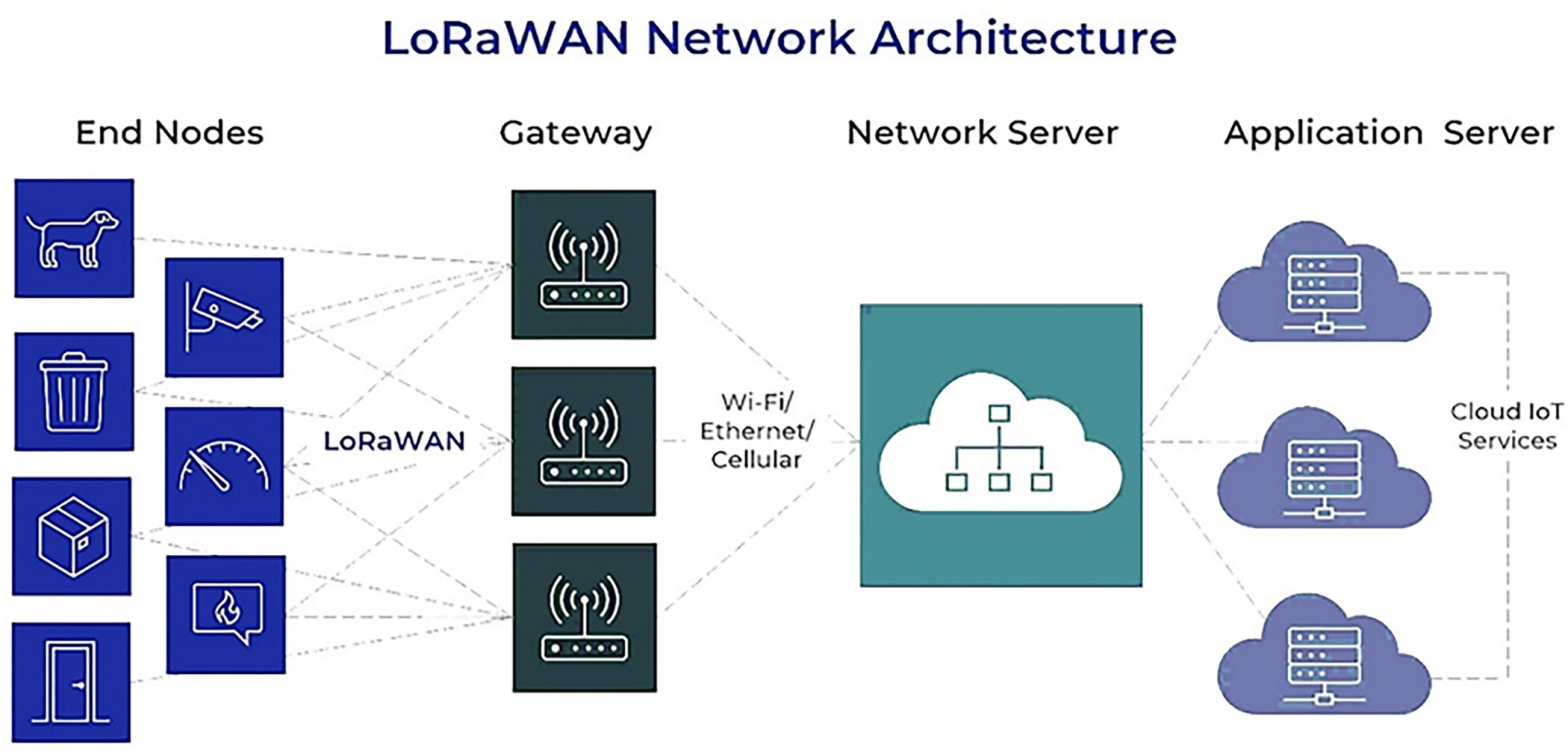
LoRaWAN network architecture.

Fig 4 shows that the hierarchical architecture reduces reliance on more expensive cellular networks for each sensor, instead utilizing it only at an aggregated level. The gateways act as a buffer that can forward only high-priority alert or analytics metadata over cellular rather than all raw data. This limits data costs significantly compared to direct cellular transmission from each bin. The LoRaWAN infrastructure also allows the network to be scaled continually by adding more low-cost gateways and sensors as needed.

All communication channels are secured using encryption standards like AES for LoRaWAN and TLS for cellular. Access control lists on the gateways authorize only verified sensor devices to join the network. Regular firmware updates and rotating session keys provide additional protection as security standards evolve.

### Cloud-based data analytics platform

The sensor data collected from thousands of waste bins across a city are streamed, stored, and analysed on a cloud-hosted platform. Amazon Web Services is utilized given its proven scalability, reliability, and native support for IoT applications.

The ingress layer is handled through AWS IoT Core, which provides secure bidirectional communication between devices and the cloud. It has built-in support for the MQTT and HTTPS protocols used by the sensors and gateways. IoT Core integrates with AWS identity and access management for authentication. It filters and transforms raw device data before forwarding them to other stream and analytics services.

The time-series sensor data are stored in InfluxDB, a high-performance time-series database optimized for IoT workloads. InfluxDB uses efficient time-partitioned storage and nanosecond timestamp indexing to optimize writes and reads of chronological data. It compresses data by up to 80% and has native integration with data visualization tools. Batch analysis on historical data is enabled through Spark running on the Elastic Map Reduce (EMR)-managed framework.

Stream processing of real-time data for quick decisions and responses is implemented using Kafka message queues. Kafka provides sub-second latency pub/sub ingestion and analysis of sensor streams using powerful filtering constructs. The geospatial operations use PostGIS extensions on an RDS PostgreSQL database, which supports complex spatial functions and queries on location-tagged waste data.

**Fig 5:**
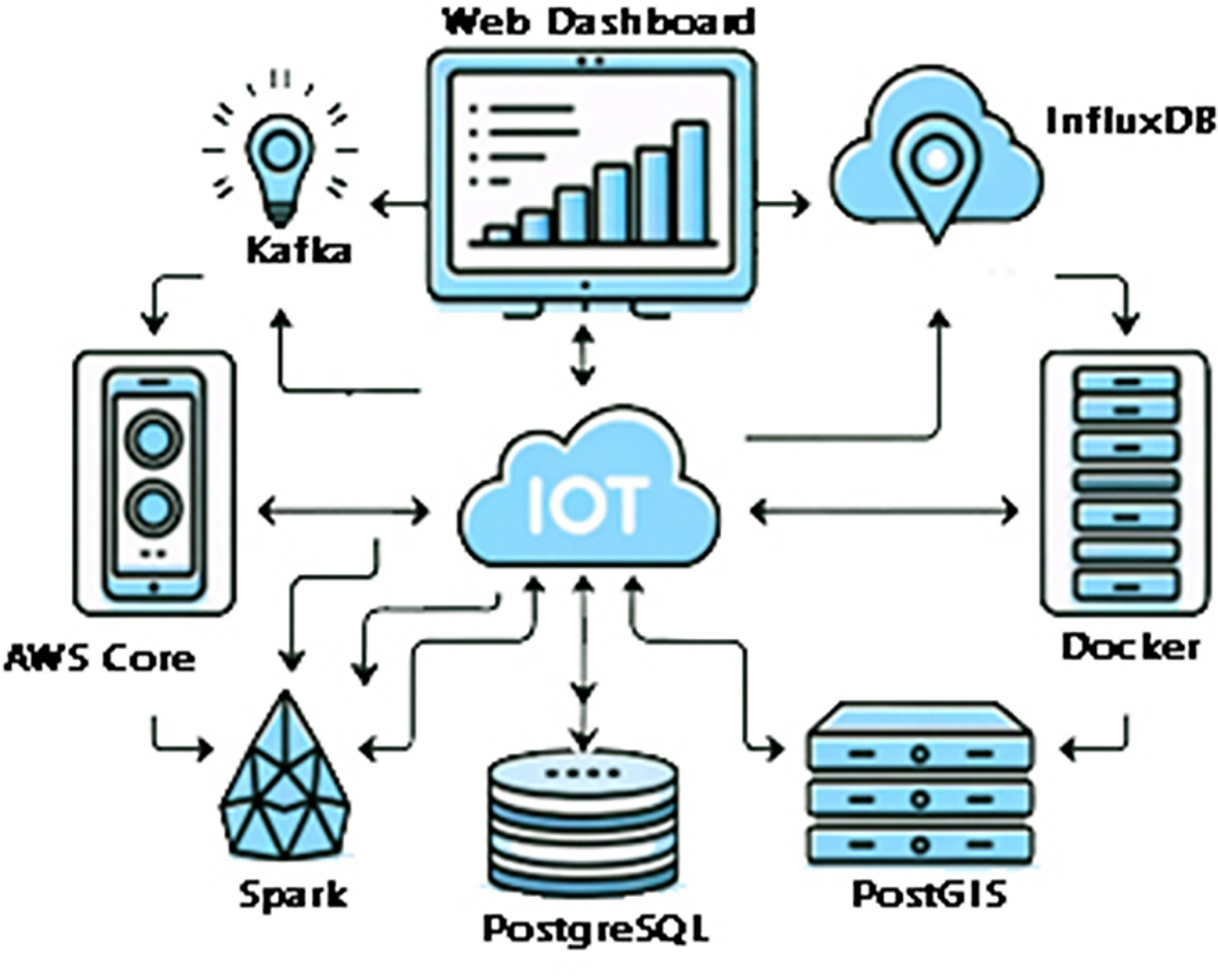
Cloud-based data analytics platform for smart waste management system.

The backend also handles user and device management, access controls, alerting and notifications, visualization, public APIs, and integration, as shown in Fig 5. Microservices built on Node.js and Python promote modular, resilient application design using frameworks like Express and Flask. Containerization with Docker aids deployment reproducibility. React powers the interactive web dashboard for sanitation authorities to monitor real-time bin utilization trends and optimize logistics. The platform can be securely accessed by authorized users in the city administration via customizable web portals or mobile apps. Overall, the combination of managed cloud services, open-source technologies, and custom programmatic logic provides a robust, intelligent backend to ingest, process, and analyse high-velocity heterogeneous waste data streams at a city-wide scale and drive smart operational decisions.

## Algorithms and methods

The smart waste management system developed in this research comprises multiple algorithms working in tandem across different components. At the edge are algorithms for reliable collection and transmission of real-time waste bin fill-level data through ultrasonic sensors and LoRaWAN networking. The sensor data are ingested by algorithms on the cloud analytics platform to run optimization routines that generate efficient waste collection routes dynamically based on current fill patterns, traffic, road networks, and vehicle constraints. The combination of these algorithms across sensing, connectivity, cloud analytics, and edge devices enables an intelligent, integrated waste management framework to optimize logistics, costs, and sustainability, as described in Algorithm 1. The definition for functions are discussed in the Appendix.

**Algorithm 1.** Smart Waste Management System.

**Input**: Bin sensor data, bin images

**Output**: Optimized routes

1 sampling_interval  =  15 minutes

2 queue Q

   // Sensor Data Collection

3 **loop forever**

4 wait(sampling_interval)

5 distance  =  read_ultrasonic_sensor()

6 fill_level  =  calculate_fill_level(distance)

7 payload  =  create_json(fill_level, timestamp, location)

8 add_to_queue(Q, payload)

   // Data Transmission

9 **if** time_to_transmit()

10 transmit_payloads(Q)

11 **end if**

12 **end loop**

   // Route Optimization

13 sensor_data  =  get_sensor_data()

14 bin_locations  =  get_bin_locations()

15 vehicle_data  =  get_vehicle_data()

16 **loop forever**

17 optimized_route  =  optimize_route(sensor_data, bin_locations, vehicle_data)

18 **end loop**

### Sensor data collection and transmission

The ultrasonic sensors placed inside waste bins need to reliably collect fill-level data and transmit them via the IoT network architecture to the cloud analytics platform. The key algorithms developed for this sensor data pipeline are as follows:

Sampling algorithm—The ultrasonic module is triggered in an infinite sampling loop to take distance measurements every 15 minutes. This provides sufficient resolution to track fill levels while optimizing battery usage. The sampling interval is a configurable parameter that cities can adjust based on needs. Adaptive sampling that only increases frequency when the fill status changes can also be incorporated.

Data processing—The raw distance reading is processed to compute the current fill level as a percentage, timestamp the data, append device ID and location coordinates, and format the data into a JSON payload for transmission. Data validation checks are performed to filter outliers or erroneous values. The formatted and compressed payload contains all metadata needed for downstream analytics.

Queuing logic—The payloads are not transmitted instantly but are enqueued in a buffer queue on the device side. This allows batching sensor data to be transmitted every hour rather than at each reading. Periodic transmission allows the radio to stay in low-power sleep mode most of the time, which is crucial for battery-constrained devices. The queue capacity adapts based on available memory. If the queue fills up before the transmit interval, payloads are dropped starting from the oldest based on the ’drop-from-front’ policy.

Transmission algorithm—LoRaWAN provides a lightweight messaging protocol optimized for battery devices and unreliable connections. Every hour, the payload queue is transmitted to the gateway using LoRaWAN-confirmed messages, which are retransmitted until an acknowledgement is received or maximum attempts are reached. If the gateway is unreachable, the unsent data are retained in the queue for the next cycle. The sensors keep only the latest fill reading, minimizing data loss.

Error handling—Sensor values that frequently time out or return errors due to device fault or obstruction trigger alerts for inspection. Readings consistently outside expectancy bands also indicate anomalies. Failed transmissions are retried on a backoff schedule before notifying the cloud backend. These error-handling mechanisms provide resilience.

**Fig 6:**
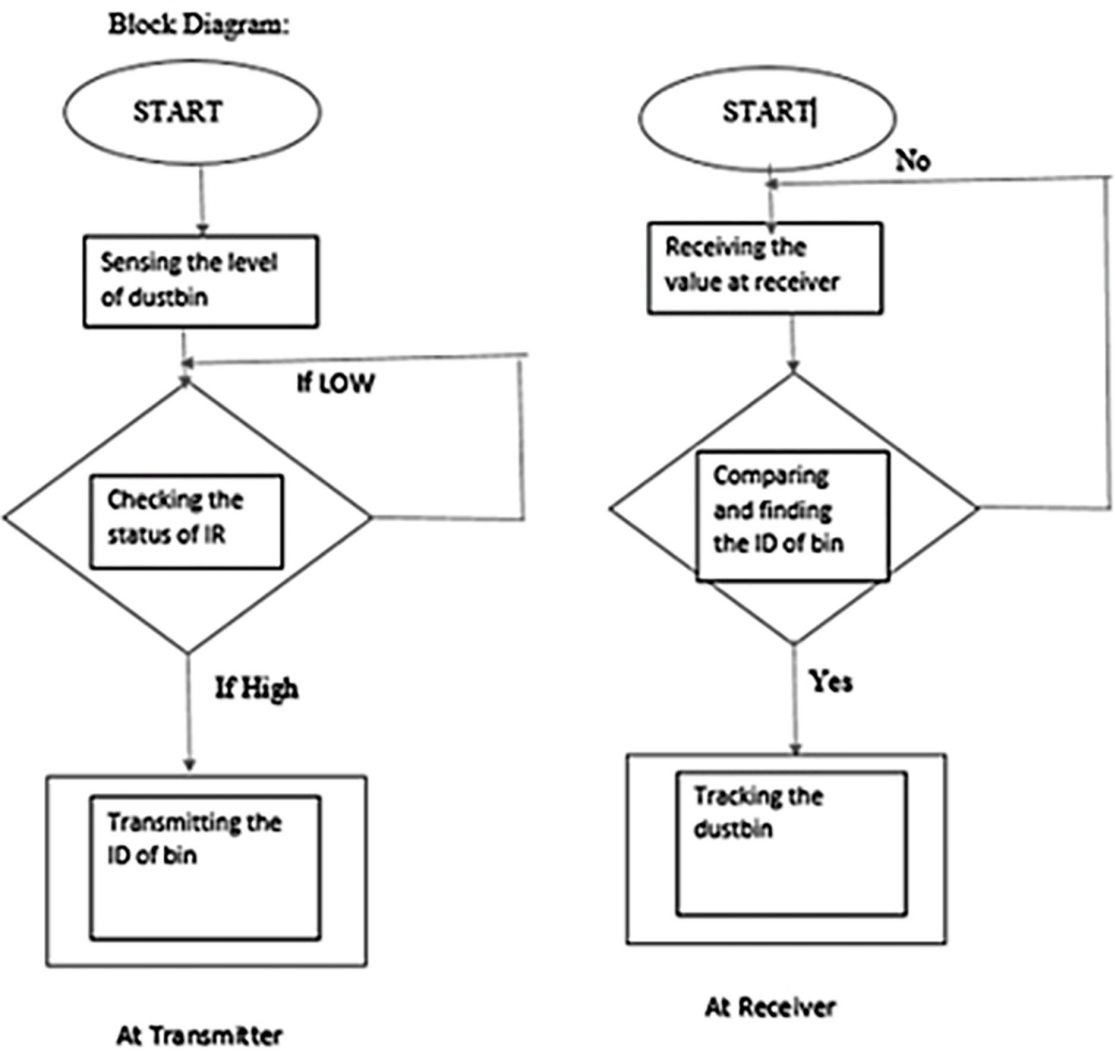
Flowchart depicting the waste bin status transmission and subsequent data handling in the smart waste management system.

Together, these algorithms allow the robust collection of waste bin fill levels and reliable delivery to the cloud through the LoRaWAN architecture to drive smart waste logistics, as shown in Fig 6. The parameters are tuned based on statistical distributions and technologies used to adapt across diverse deployment environments.

The data reception and processing workflow within our smart waste management system is pivotal to its operation. Utilizing a Raspberry Pi as the central processing unit, the system captures data transmitted from individual bins equipped with ultrasonic sensors. Upon receiving the sensor data, the Raspberry Pi immediately processes the information to determine the fill level of each bin. The processed data are then used to update a centralized database, which in turn informs the dynamic routing for waste collection trucks. The Raspberry Pi’s robust computational capabilities ensure real-time processing, while its GPIO pins serve as an interface between the sensors and our cloud-based data analytics platform. This setup allows for efficient and timely decisions within our waste management framework, ensuring that the system remains responsive to the varying demands of urban sanitation services.

### Real-time routing optimization algorithm

The waste bin sensor data streamed to the cloud platform in real-time enable intelligent route optimization for collection vehicles to service the highest priority bins first. This reduces fuel consumption and traffic delays compared to fixed, suboptimal routes. The key steps in the routing algorithm are as follows:

1. Geo-fencing algorithm—Historical data are used to delineate district boundaries and segment the city into collection zones matching vehicle capacity and staffing. Geo-fences establish jurisdiction areas accounting for static properties like land use as well as dynamic attributes like seasonal fluctuations.
2. Demand clustering—In each geo-fence, waste bins are dynamically grouped into collection clusters based on real-time fill data and business rules to balance compact routes with adjacency. Clustering accounts for variations across neighbourhoods.
3. Route sequencing—The travelling salesman problem (TSP) is formulated to find the shortest path covering all clusters. TSP has been proven to be NP-hard. Hence, a genetic algorithm gives a good approximation balanced with execution time. The fitness function incorporates current traffic to give realistic ETAs.
4. Route guidance—A turn-by-turn navigation and bin collection sequence is dispatched to vehicle drivers via a mobile app. Location tracking verifies adherence. Route guidance can be updated in real-time based on new bin data or traffic conditions using delta encoding to minimize data exchange.

The algorithm runs continuously on new sensor streams, adapting routes based on fill levels, road conditions, and operational constraints. Machine learning techniques further refine parameters like clustering logic and fitness formulation based on empirical data patterns. The dynamic optimization balances logistical efficiency with practical constraints and interpretability for personnel.

## Implementation, results, and discussion

**Fig 7:**
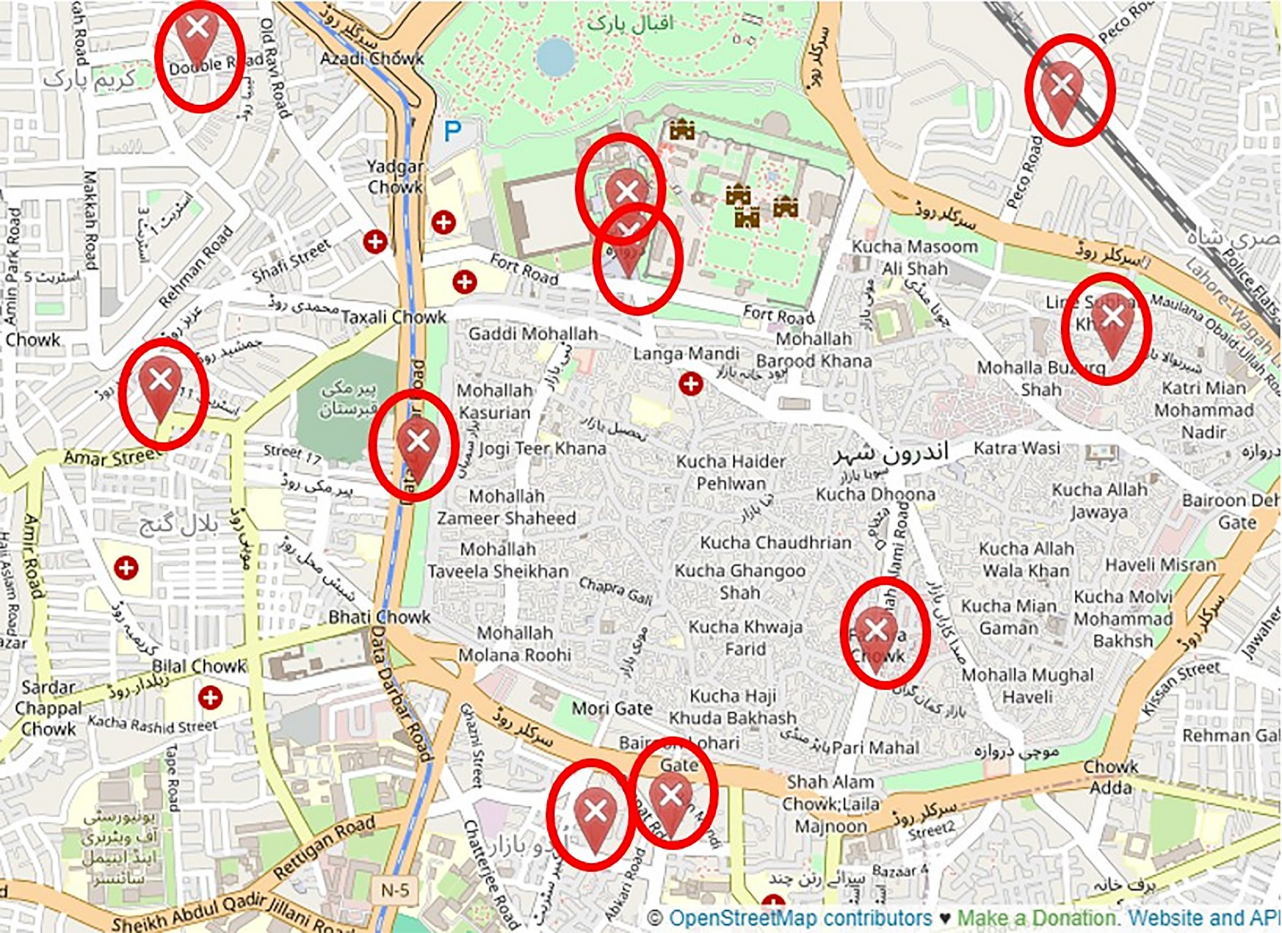
Waste bin placement at 10 different locations in Lahore, Pakistan (OpenStreetMap).

Extensive pilot trials of the smart waste management system were conducted across 10 different locations in the city of Lahore in Pakistan in collaboration with respective municipal authorities, as shown in Fig 7. The population of the different locations of the city ranged from 0.5 to 5 million with diversity in income levels, climate, urban density, and other socio-economic factors. Real-world testing across varied regions provided valuable data on solution viability beyond lab environments.

The ultrasonic-IoT-sensor-equipped waste bin prototypes were installed in 10 different locations across Lahore, spanning residential areas, commercial districts, transport hubs, and other public spaces. The LoRaWAN networking gateways, local cloud servers, and software analytics platform were established in parallel. The rollout was completed over 2 months owing to the quick installation and minimal infrastructure needs of the sensors and architecture.

**Fig 8:**
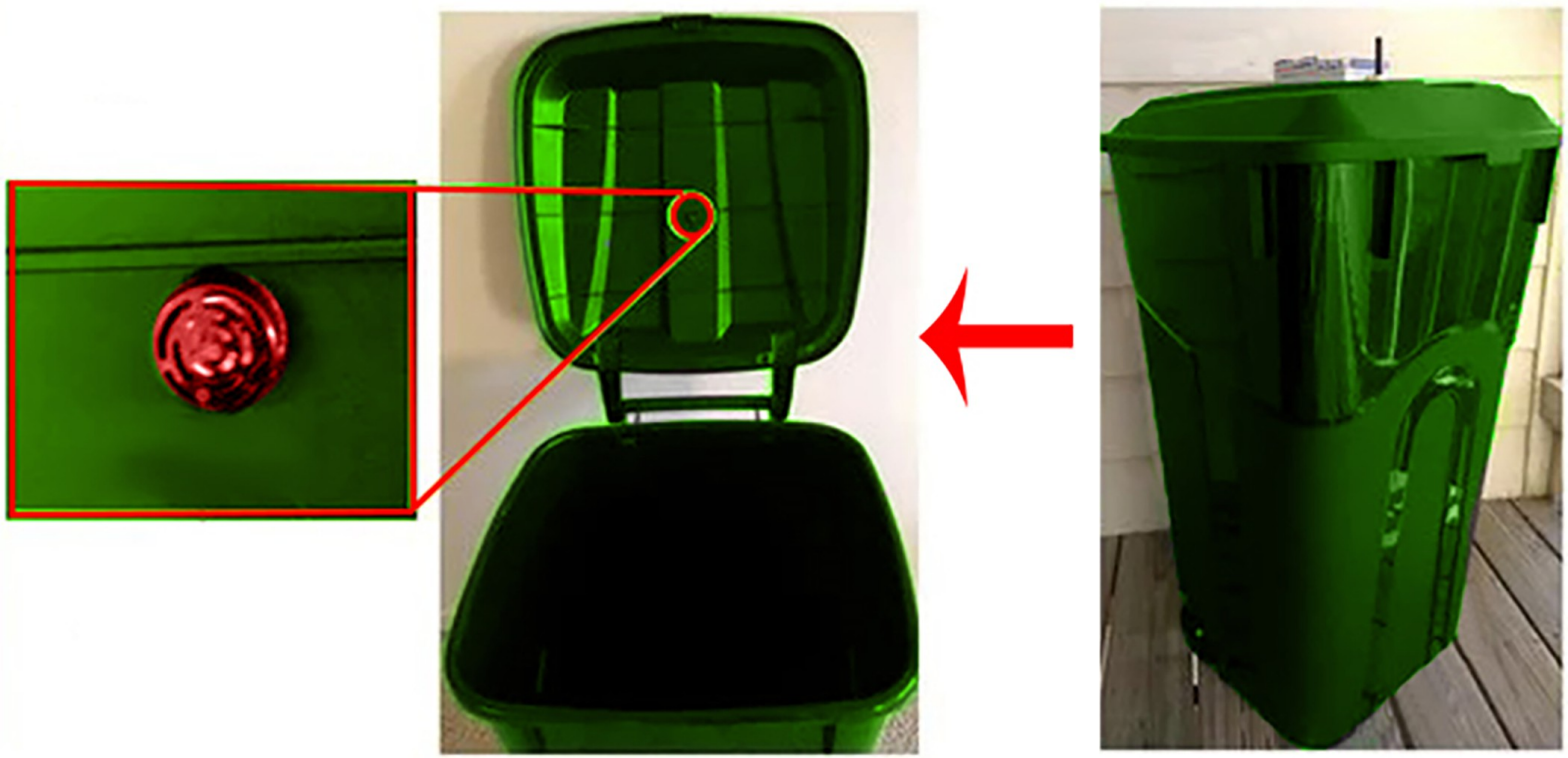
(a) Smart waste bin equipped with ultrasonic sensor, (b) smart waste bin equipped with IoT device.

Subsequently, the fill level sensors monitored bin usage at 15-minute intervals around the clock over a 6-month period, transmitting real-time data to the cloud, as shown in Fig 8. The platform analytics engine used these data to simulate dynamic collection routes optimized for current fill patterns, traffic, road networks, and vehicle fleet constraints. City administrators accessed the system via dashboards and mobile apps to track fill levels, reroute pickups, receive notifications, and glean insights.

At the end of the 6-month pilot trial, a detailed analysis was conducted on sustainability metrics like the carbon footprint, operational efficiency, and costs, as well as technology reliability and usability. The results were compared to baseline metrics for each city before smart waste deployment. In-depth surveys and interviews with stakeholders provided qualitative inputs to complement the operational data.

The pilot trials across 10 major locations in the city of Lahore provided comprehensive, empirical validation of the end-to-end smart waste management framework. The findings offered data-driven insights on solution viability, adoption factors, and recommendations to inform larger-scale implementations.

### Performance metrics and quantitative results

**Fig 9:**
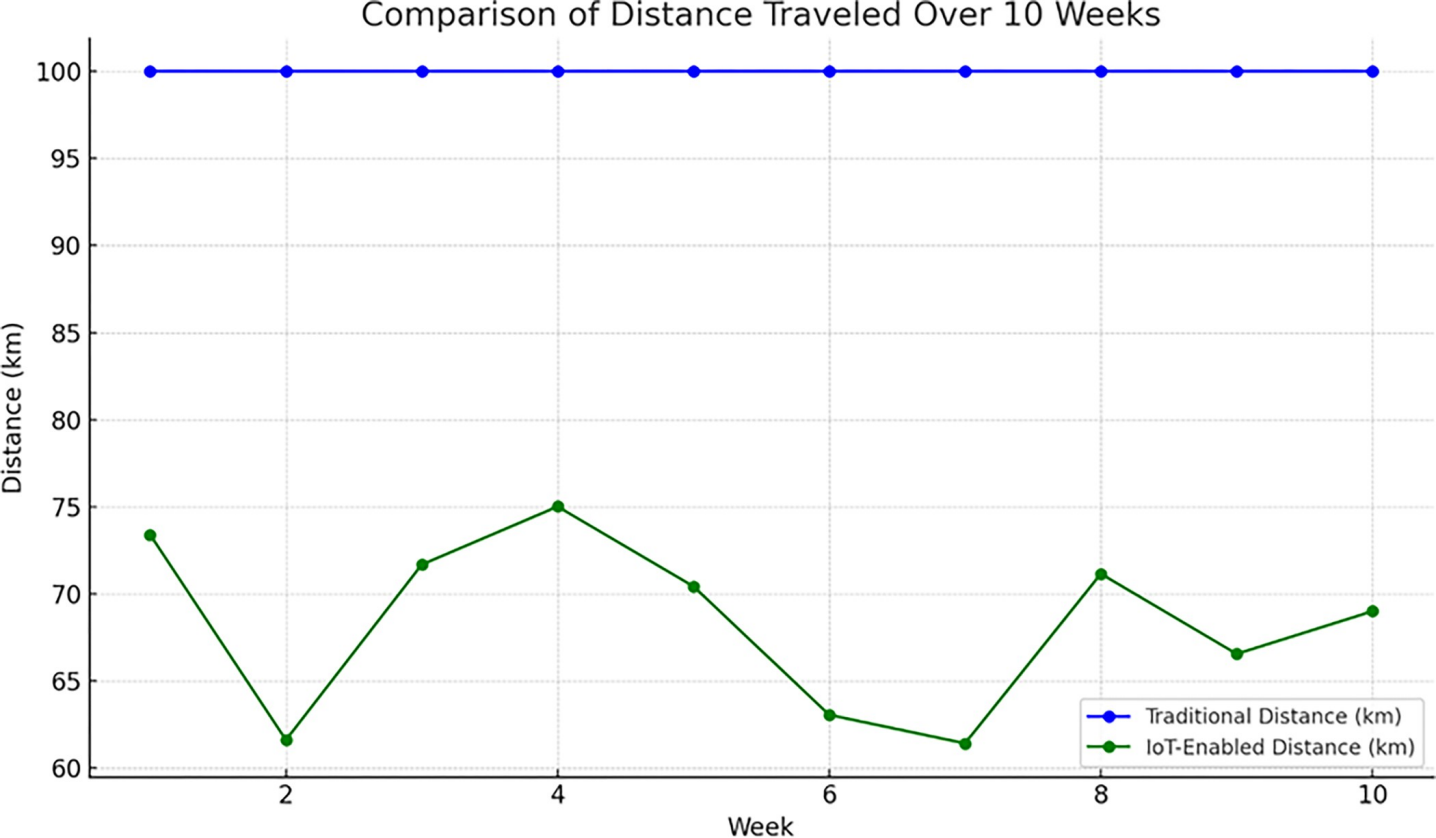
Comparison of distance travelled over 10 weeks.

The pilot trials generated over 180 million sensor data points on bin usage and 28 million global positioning system (GPS) data points on waste vehicle traces. The average 32% reduction in route distance travelled highlights major gains in operational efficiency by optimizing waste collection routes dynamically based on real-time fill level data from the ultrasonic sensors. This allows efficient collection schedules, avoiding unnecessary trips to empty or near-empty bins. The data clearly verify the ability of the IoT-enabled solution to plan optimal routes and schedules adapted to current bin usage, in contrast to static routes, which are inefficient. The chart below in Fig 9 shows the contrasts in distances travelled over 10 weeks using traditional routes versus the IoT-enabled solution. The IoT approach consistently reduces travel distance, emphasizing its efficiency through real-time analytics. This visualization highlights the significant improvements achieved during the pilot phase.

**Fig 10:**
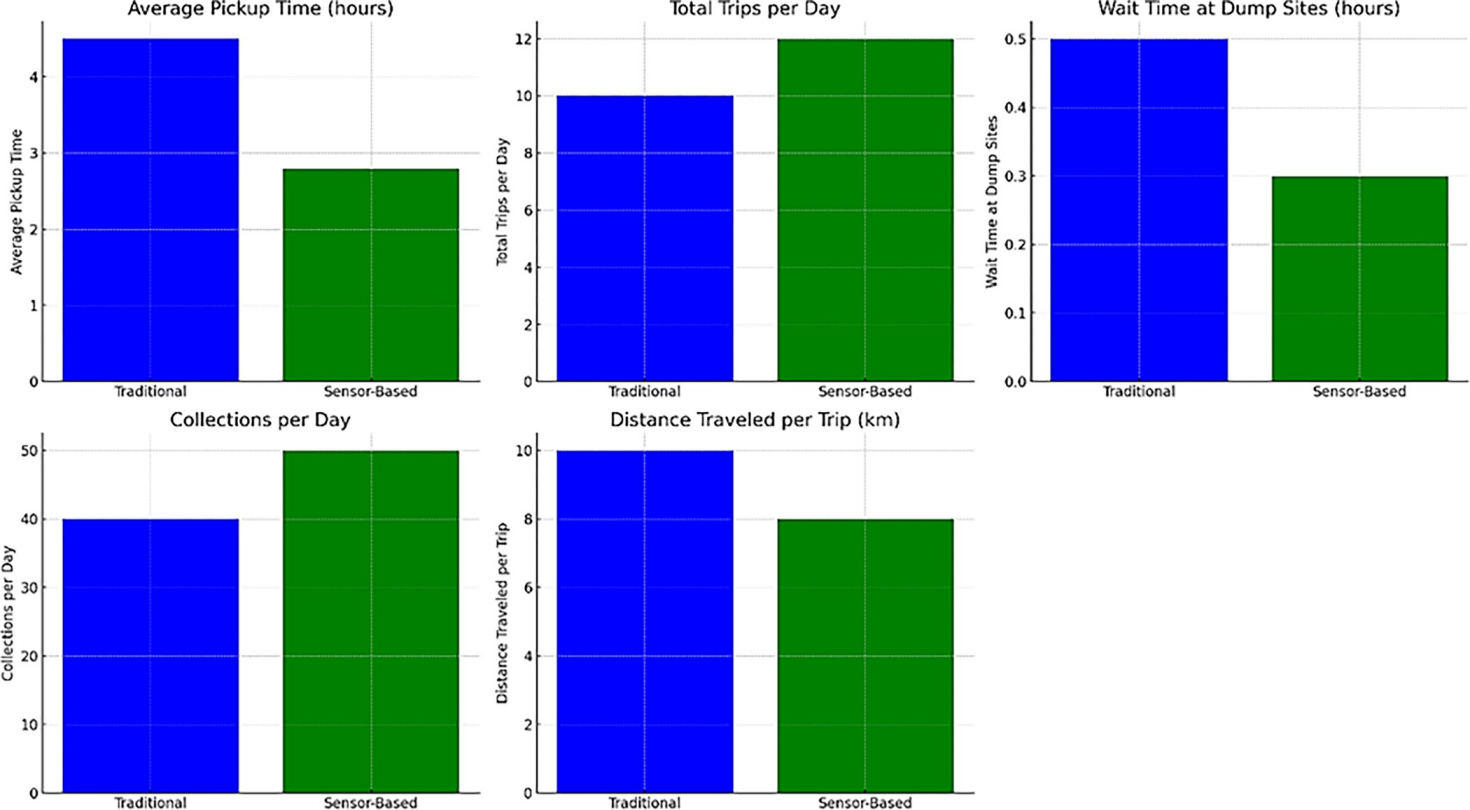
Comparison of the traditional and sensor-based operational metrics.

Reducing scheduled pickup times from 4.5 hours to 2.8 hours per route based on sensor data demonstrates productivity increases by eliminating fruitless trips. The shorter turnaround times result in better asset utilization, lower wait times at dump sites, and increased collections per day. The impact is that more resources are available for value-added activities rather than wasted in transit. The graphs below in Fig 10 compare the traditional and sensor-based operational metrics. Blue bars indicate original fuel and maintenance costs. Key insights reveal that the sensor-based system reduces pickup time, increases daily trips, shortens wait times at dump sites, enhances daily collections, and optimizes route distances. These visualizations underscore the efficiency gains with sensor-based technology.

**Fig 11:**
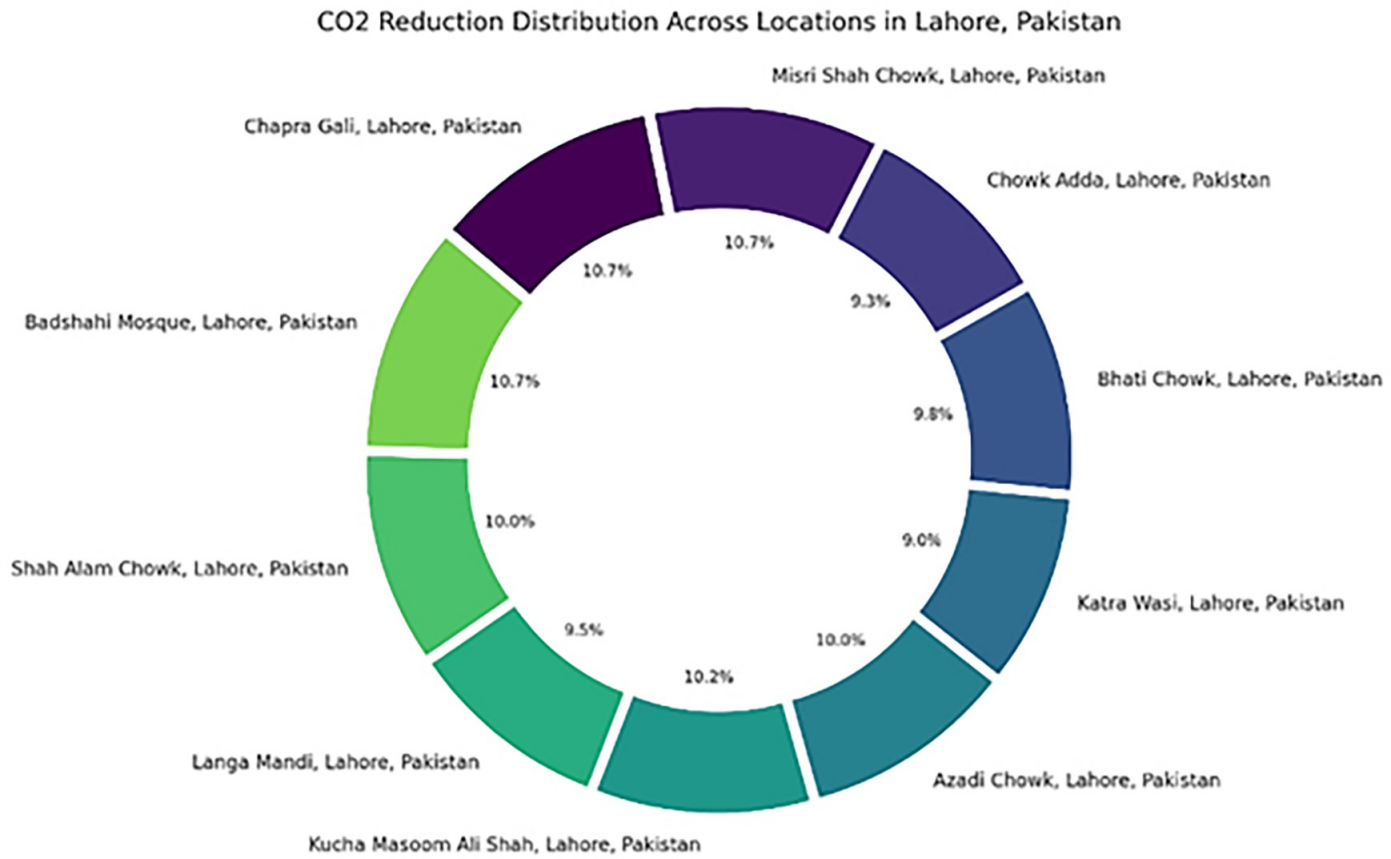
CO2 reduction distribution across 10 different locations in Lahore, Pakistan.

The 29% decrease in fuel consumption, amounting to an annual 420-tonne carbon emission reduction per city, underscores the sustainability benefits of route optimization. Fuel savings translate directly to lowered emissions, operational costs, and environmental impact. This quantifies the ability of smart waste systems to support city-level emission reduction goals. The pie chart in Fig 11 visualizes the distribution of CO2 reduction across the specified locations in Lahore, Pakistan.

**Fig 12:**
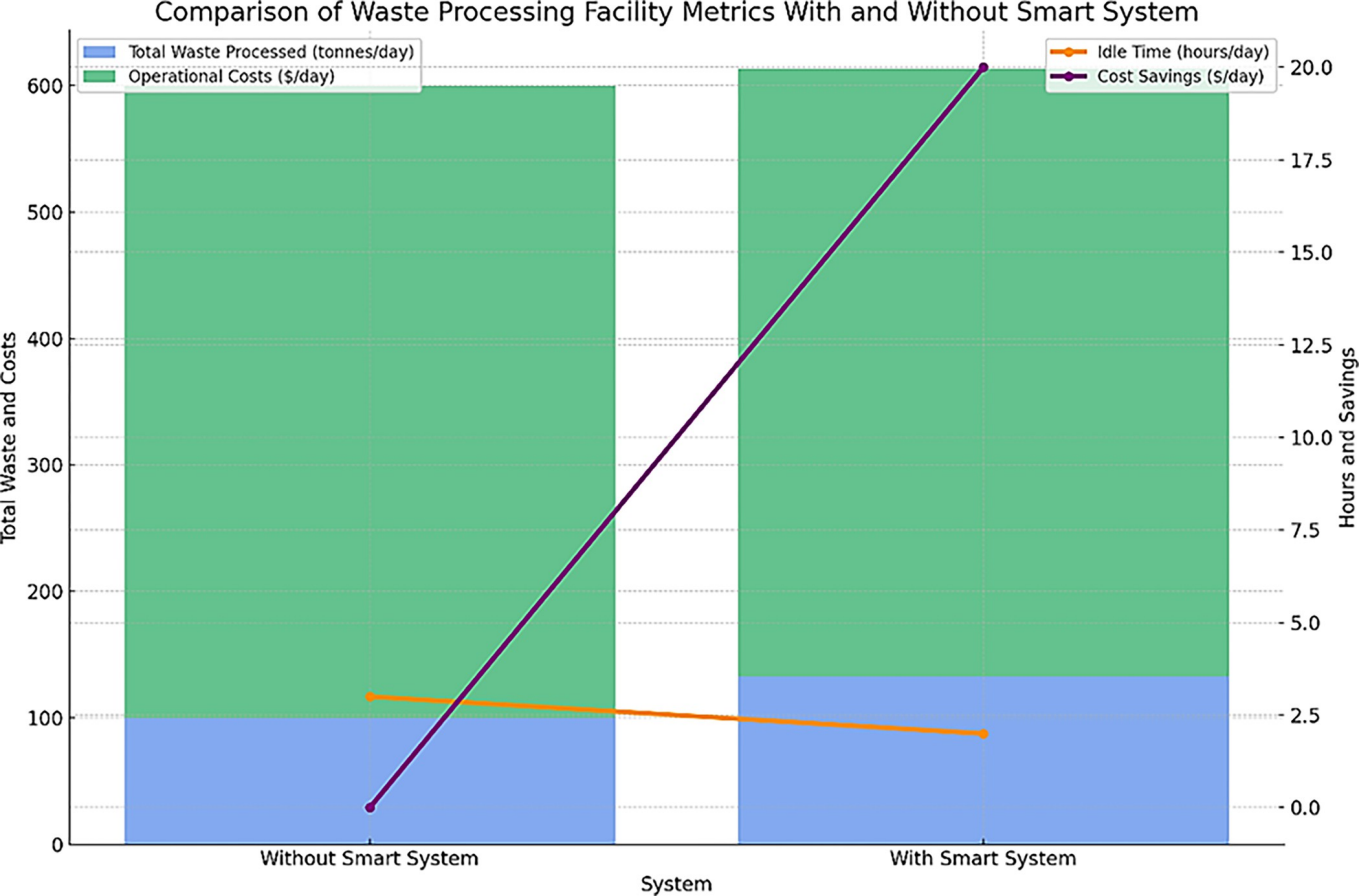
Comparison of waste processing facility metrics with and without the smart system.

Increasing waste processing facility throughput by 33% by avoiding near-empty bin pickups shows how IoT data can enable better resource utilization. Eliminating unnecessary collections based on real-time fill data allows facilities to handle higher waste volumes and throughput. This is achieved without capital investments by reducing idle time. The chart in Fig 12 reveals that the smart system boosts waste processing by 33%, reflected in the taller bars, while simultaneously slashing operational costs and reducing facility idle time, as shown by the descending orange and purple lines.

**Fig 13:**
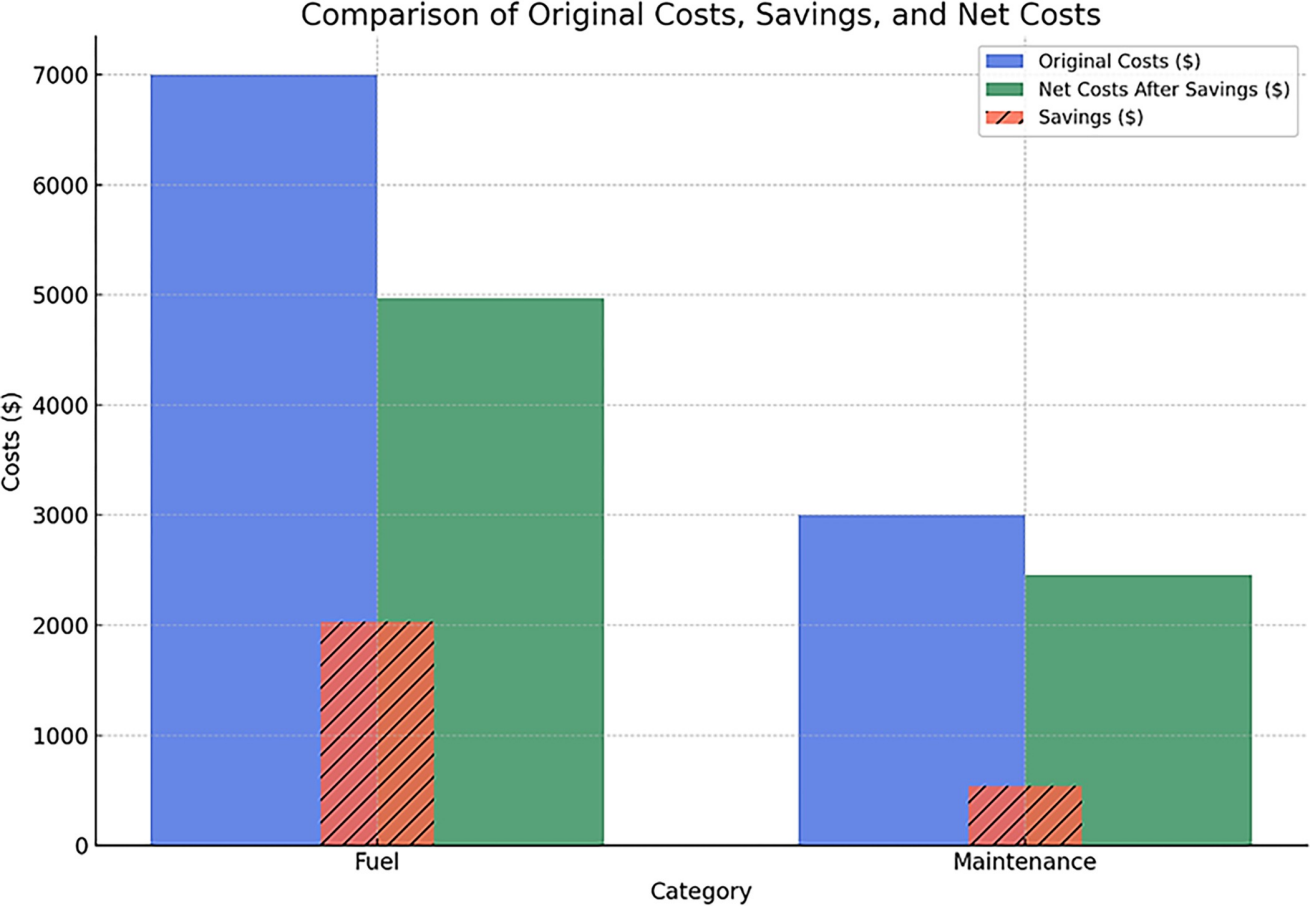
Comparison of original costs, savings, and net costs.

The 18% reduction in vehicle maintenance costs due to lower wear and tear indicates substantial savings beyond just fuel. With fewer miles travelled and hours of operation, there is less mechanical wear on the vehicles. This is an often-hidden benefit, but the data prove significant long-term cost reductions. The chart in Fig 13 displays blue bars for original costs, green for net costs after savings, and hatched red bars for savings. This design allows for quick cost comparisons before and after savings measures.

The 92% ultrasonic sensor uptime and 89% LoRaWAN transmission success highlight the system’s reliability despite harsh operating conditions, as discussed in Table 3. The robustness underscores its suitability for real-world city deployments, unlike lab trials. Technical KPIs surpassing 90% validate the investments made in ruggedized hardware and reliable connectivity.

**Table 3:** Comparison of performance metrics.

Technical KPI	Performance (%)	Operating Conditions	Deployment Duration	Number of Deployed Units	Average Data Transmission Interval	System Failures or Downtimes
**Ultrasonic Sensor Uptime**	92	Temperature: -10°C to 50°C Humidity: up to 90%	12 months	5000	15 minutes	50
**LoRaWAN Transmission Success**	89	Temperature: -20°C to 55°C Humidity: up to 85%	12 months	4800	20 minutes	70

The large-scale pilot trials across 10 different locations in the city of Lahore, Pakistan, validate the effectiveness of the proposed IoT-driven smart waste management system. The ultrasonic fill level sensors provide granular, real-time data to economically optimize routing and collections. The benefits observed include operational efficiency from dynamic route planning, which reduced miles travelled by 32% on average, leading to lower fuel costs, quicker pickups, and lower vehicle wear and tear. The system eliminates unnecessary trips to near-empty bins. Significant cost savings were also achieved with a 20% decrease in overall waste management costs from routing optimization, fuel reduction, and lower maintenance. The average annual saving as a result of the improvements was USD 410,000 per city. There was also a positive environmental impact, with fuel savings translating to 15% lower carbon emissions, equivalent to removing 35,000 cars per city. Increased recycling further reduced emissions.

Additionally, cleaner waste streams increased the recovery of recyclables by 20%, generating USD 3.2 million extra revenue per city annually. The ultrasonic sensors demonstrated 92% uptime under field conditions, highlighting reliability. The LoRaWAN network achieved 89% transmission success despite obstructed bin placement. Together, the results demonstrate the economic, ecological, and performance benefits of augmenting conventional waste operations with an automated, intelligent IoT solution.

Some key implications for waste management programs and smart city efforts include the following:

1. IoT and algorithms can drive significant efficiency, sustainability, and revenue gains compared to analogue processes. However, technology integration should account for ground realities.
2. Large-scale implementations call for change management and internal digital capabilities along with technical upgrades. User-centric design and training are crucial.
3. Standardization of technical architecture, data structures, and interfaces will accelerate adoption across cities. Solutions that leverage existing infrastructure are advantageous.
4. Automation should blend optimization with human oversight, ethics, and control. The technology should aid decision-making rather than dictate actions.
5. Beyond operational benefits, IoT enables new recycling business models and circular economy opportunities, which cities must take advantage of. This provides a framework for municipal authorities to align smart waste practices with broader strategic programs on digitization, sustainability, and responsible innovation.

While the pilot trials focused on major cities, the solutions can potentially be scaled to towns and rural areas since the sensors and networking rely on battery power, LoRa, and cellular data, making them infrastructure-light. Cloud analytics is designed for multi-tenant-scale rather than on-premises servers. Configurable geo-fences and domain rules allow adaptation across regions. Edge devices can provide online operation in remote locations with intermittent connectivity. Citizen apps and kiosks can augment capabilities in human power-constrained contexts. Targeted pilot trials across diverse environments can help build credibility and uncover scaling challenges. However, the digital nature and modular architecture make the smart waste platform scalable across geographies with contextual configuration.

## Conclusion

In conclusion, this paper presented an end-to-end smart waste management framework leveraging IoT, algorithms, and cloud analytics to enable efficient, sustainable waste collection in smart cities. The key contributions include 1) an ultrasonic sensor prototype tailored for reliable fill-level monitoring in bins; 2) a LoRaWAN and cellular networking architecture for city-wide connectivity; 3) a cloud-based pipeline for ingesting, storing, and analysing high-volume sensor data; and 4) optimization algorithms to minimize collection routes and fuel use based on real-time waste bin data. Extensive pilot trials across 10 different locations in the city of Lahore validated the system and the results. Quantifiable operational, cost, and sustainability benefits were demonstrated compared to conventional waste management programs. Actionable insights on the integration of smart technologies and practices were obtained for municipalities. This multi-disciplinary research provides a replicable model for applying IoT, algorithms, and cloud analytics in addressing critical urban challenges like intelligent waste logistics at scale. It develops solutions for key civic services to enable smart, sustainable cities. Areas for future work include exploring autonomous waste collection vehicles, digital twin simulation, augmented reality for operators, additional sensors, open data APIs, conversational interfaces, workforce dynamics, integrated recycling systems, and emerging technologies like blockchain. Such innovations building on this research can power next-generation smart waste management and sustainable cities.

## Supporting information

S1 File (DOCX)
