# Supplementary material for: Waste management 2.0 leveraging internet of things for an efficient and eco-friendly smart city solution
Source: PLoS One. 2024 Jul 31;19(7):e0307608. doi: 10.1371/journal.pone.0307608 (PMC11290616; doi:10.1371/journal.pone.0307608)
Supplement: S1 File — (DOCX) [file pone.0307608.s001.docx]

Annexure

Complexity Analysis and Function Definitions

Complexity of Algorithm 1

Algorithm 1 is designed to process a dataset efficiently. To understand its performance, we need to analyze its computational complexity, which provides insights into the algorithm’s efficiency, especially as the size of the dataset grows. The analysis is broken down into the following key components:

1. **Initialization**:
   - **Description**: This step involves setting up initial variables, constants, and data structures required by the algorithm.
   - **Complexity**: Since initialization typically involves assigning values and allocating memory, which does not depend on the size of the input dataset, its complexity is $O(1)$.
2. **Main Loop**:
   - **Description**: The main loop iterates over the dataset elements. If the dataset has nnn elements, the loop runs nnn times.
   - **Complexity**: If each iteration of the loop performs operations that take $O(f(n))$ time, then the total complexity contributed by this loop is $O(n\cdot f(n))$.
3. **Inner Operations**:
   - **Description**: Inside the main loop, there may be nested loops or function calls that perform specific tasks on each data element.
   - **Complexity**: If these inner operations include a nested loop or a function that processes each element in $O(g(m))$time, where mmm could be related to nnn or independent, the overall complexity of these operations is $O(n\cdot g(m))$.

Combining these components, the total complexity of Algorithm 1 can be expressed as:

$$O(1)+O(n\cdot f(n))+O(n\cdot g(m))$$

Since $O(1)$ is constant and does not significantly affect the total complexity for large nnn, it can be simplified to:

$$O(n\cdot f(n)+n\cdot g(m))$$

To make this more concrete, let's consider a specific example where $f(n)=n$ (i.e., a linear operation within the main loop) and $g(m)=log(m)$g(m) (e.g., a logarithmic operation within a nested function). In this case, the total complexity would be:

$$O(n\cdot n+n\cdot log(m))=O(n^{2}+nlog(m))$$

# Definitions for Functions Used in Algorithm 1

To provide a comprehensive understanding, we need to define the functions used within Algorithm 1. This helps in understanding their individual complexities and how they contribute to the overall complexity of the algorithm.

1. **Function initializeVariables**:
   - **Definition**: This function is responsible for setting up initial variables and data structures. It may include initializing counters, setting up arrays or lists, and other preparatory steps.
   - **Example Code**:

def initializeVariables():

counter = 0

data_structure = []

# Additional initializations

- - **Complexity**: The operations in this function are constant with respect to the size of the input data. Therefore, its complexity is $O(1)$.

1. **Function processData**:
   - **Definition**: This function is called within the main loop to process each element of the dataset. The specific operations can vary but often include computations, data transformations, or conditional checks.
   - **Example Code**:

def processData(element):

# Perform some operations on the element

result = element * 2 # Example operation

return result

- - **Complexity**: Assuming the operations inside processData are $O(1)$, the complexity of processing each element is $O(1)$. Therefore, for nnn elements, the total complexity is $O(n)$.

1. **Function nestedOperation**:
   - **Definition**: This function represents any nested operations that might be called within the main loop. These could be more complex functions involving additional loops or recursive calls.
   - **Example Code**:

def nestedOperation(element):

for i in range(len(element)):

# Perform some operations

element[i] = element[i] * 2

return element

- - **Complexity**: If the nested loop iterates mmm times for each element, and each iteration is $O(1)$, the complexity is $O(m)$ per element. For $n$ elements, it becomes $O(n\cdot m)$.

1. **Function finalizeResults**:
   - **Definition**: This function is called after the main loop to finalize and prepare the results. It might involve aggregating data, formatting the output, or performing final checks.
   - **Example Code**:

def finalizeResults(data):

# Aggregate or format data

result = sum(data) # Example operation

return result

- - **Complexity**: Similar to initialization, the finalization step usually involves a fixed number of operations, making its complexity $O(1)$.
